# 'Diversion’ of methadone or buprenorphine: 'harm’ versus 'helping’

**DOI:** 10.1186/1477-7517-10-24

**Published:** 2013-10-16

**Authors:** Ingrid Amalia Havnes, Thomas Clausen, Anne-Lise Middelthon

**Affiliations:** 1 Division of Mental Health and Addiction, Oslo University Hospital, Nydalen, PO Box 4956, Oslo 0424, Norway; 2 SERAF - Norwegian Centre for Addiction Research, University of Oslo, PO Box 1039, Oslo 0315, Norway; 3 Institute of Health and Society, University of Oslo, Blindern, PO Box 1130, Oslo 0318, Norway

**Keywords:** Diversion, Opioid maintenance treatment, Methadone, Buprenorphine, Reciprocity

## Abstract

**Background:**

'Non-compliant’ individuals in opioid maintenance treatment, OMT, are often met with tight control regimes to reduce the risk of 'diversion’, which may lead to harm or death among persons outside of OMT. This article explores reported practices of, and motivations for, diversion of methadone and buprenorphine, in a group of imprisoned individuals in OMT.

**Findings:**

28 in-depths interviews were conducted among 12 OMT-enrolled, imprisoned individuals, most of whom were remand prisoners. All had experienced tight control regimes prior to imprisonment due to varying degrees of 'non-compliance’ and illicit drug use during treatment. Their acquired norm of sharing with others in a drug using community was maintained when entering OMT. Giving one’s prescription opioids to an individual in withdrawal was indeed seen as an act of helping, something that takes on particular significance for couples in which only one partner is included in OMT and the other is using illicit heroin. Individuals enrolled in OMT might thus be trapped between practicing norms of helping and sharing and adhering to treatment regulations. ’Diversion’, as this term is conventionally used, is not typically understood as practices of giving and helping, but may nevertheless be perceived as such by those who undertake them.

**Conclusions:**

As we see it, the need to sustain oneself as a decent person in one’s own eyes and those of others through practices such as sharing and helping should be recognized. Treatment providers should consider including couples in which both individuals are motivated for starting OMT.

## Background

'Non-compliant’ individuals in opioid maintenance treatment, OMT, are often met with tight control regimes [1-3] to reduce the risk of 'diversion’ and thereby prevent harm or death among persons outside of the treatment program [4]. Reported motivations among persons who divert OMT medications consist of selling to support one’s own economy [5], as well as *giving* to friends and acquaintances as a social resource [6,7]. Thus, a thorough understanding of the realities of the people such measures are meant to meet needs to be internal to the planning, development and implementation of treatment regulations in OMT. This article, which is based on a study that explored motivations for criminal activity, focuses on reported practices of and motivations for methadone and buprenorphine diversion in a group of imprisoned, OMT-enrolled individuals.

## Context

The Norwegian OMT programme started in 1998 as a restrictive and high-threshold treatment system [8]. In 2004, individuals in OMT obtained rights as patients. The 2010 national guidelines focus on individual rehabilitation, patient rights and harm reduction, at personal and societal levels. Individuals lacking or with positive urine tests may be subject to daily, supervised intake of OMT medication and may be limited in their medication choice. The guidelines emphasize the importance of social network mapping, with a focus on possible substance use. Though it is not an explicit goal, couples can be in treatment together [9].

## Sample and method

The findings presented in this paper are derived from a qualitative study that formed part of a larger crime study [10-12]. All together, 28 semi-structured interviews were conducted with twelve imprisoned individuals between 22 and 50 years of age; nine men and three women. The majority of these participants were remand prisoners and ten were formerly convicted of violent crime. Time previously served in prison ranged 1.5 to 20 years. All interviews were conducted in prison. For cross-case analysis and to validate findings, repeat interviews were performed for all but two participants who were released from the remand wing on short notice. Among the interview topics explored were: experiences with OMT, diversion of OMT-medications, norm systems, health issues, motivations for and understandings of criminal activity during OMT and life situations before imprisonment. The interviews were audio recorded and transcribed verbatim. The exploratory, thematic analysis was carried out by the first and last authors, with a reflexive and interactive approach throughout the entire research process.

### Ethics

Ethical approval was obtained from the Norwegian Regional Committee for Medical Research Ethics, the Norwegian Social Science Data Services and the Norwegian Correctional Service Region East. Verbal and written consent procedures were carried out with all participants. Emphasis was placed on ensuring anonymity throughout the publication process.

## Findings

Those who participated in this study had all been convicted of theft and drug-related crimes. The majority were also convicted of violence towards others, thus exhibiting what is often regarded as 'anti-social’ behavior [13-15]. But, so-called 'anti-social’ behavior can hardly be seen as the *only* form of sociality demonstrated. Practices of and attitudes towards helping and giving were hence among the phenomena explored in our endeavor to achieve a fuller account of the social lives of these imprisoned, opioid-dependent persons.

While only one of the project participants reported regularly selling or exchanging his methadone for heroin, several individuals had indeed developed strategies to prevent themselves from selling their prescription opioids. Among these were: avoiding potential buyers by taking alternatives routes to and arriving late at the pharmacy, maintaining a secretive status as OMT-patients and asking family members for help with monetary problems. Those who gave methadone and buprenorphine to friends and acquaintances regarded doing so as 'helping’ and 'giving’, as opposed to selling or exchanging. They all experienced tightly-controlled opioid prescription regimes outside of prison as a hindrance to 'helping’ others in need. 'Helping’ had been possible, however, and especially for those who received one take-home dose on weekends.

In what follows, we present some cases. We begin with Hugo and Ståle, both of whom had unstable housing and lived in homeless shelters prior to and during OMT, hence experiencing daily contact with friends and acquaintances in active heroin use. Hugo was among those who appreciated being able to help others. He explained that, due to daily supervised intake of buprenorphine, he had only been able to help a close friend a few times while enrolled in OMT. He contrasted this with a two-year period prior to OMT when he had used illegal buprenorphine on a daily basis. At that time, he had access to large amounts of buprenorphine, making it possible to regularly give it to a friend in withdrawal. Hugo did not want anything in return. Ståle, who occasionally gave his stockpiled methadone to a friend in withdrawal was also clear about the fact that he did not expect anything in return. It should be noted that such acts of 'helping’ take on particular significance for couples. Erik, for example, lived together with a woman addicted to heroin for many years prior to OMT. He explained that they were then mutually responsible for obtaining heroin:

One day one will manage to get some [heroin], the other day the other will manage to get some, or we will get some together. Or we don't get any. You don’t always have some.

While Erik accepted that he could not always obtain heroin, it was impossible for Mona to do so. She strongly feared withdrawal and said that she needed a steady income to ensure that she could always buy the heroin she needed. When she became involved in a relationship with a heroin-dependent man, higher income was needed. Even if he contributed, Mona did not have the security that she needed and explained that her choice was then between 3 “hells”: selling drugs, committing property crime or selling sex. She chose the latter because it gave her more control. Her male partner was included in OMT due to his worsening health status. He received a daily, supervised dose of methadone. Hence, he could not share his methadone when she was in withdrawal. They lived together, he in OMT and she on heroin. She continued to sell sex and regularly experienced violence and humiliation. This was a painful situation for both.

In contrast to Mona and her partner, Simon was among those who found a situation in which only one partner was enrolled in OMT to be an impossible one. Before entering treatment, he had had a partner who was addicted to heroin and was clear about his opinion that a relationship in which one partner is in treatment and one dependent on illegal drugs involves unsolvable and unbearable moral dilemmas:

Then you have to come up with ways [to give away your medication] to help your girlfriend if she is having a bad day. You can't just leave your girlfriend in withdrawal if you can help. So you're off to hell of a bad start if you can't both get help [treatment], together.

## Discussion

The study participants had all committed serious crimes and reported criminal activity during OMT. They were all imprisoned at the time of the interviews, which may have influenced their decisions to share particular experiences and events and perhaps also their understandings of and retrospective reflections on these events and their consequences [16]. All participants were seen as 'non-compliant’ by treatment providers and were subject to daily, supervised intake of their opioid prescriptions outside of prison. Nonetheless, these facts should not be taken to imply that they were exclusively 'anti-social’ [13-15]. Social dimensions are often unaccounted for when encoding 'deviant behavior’ [17,18] and it seems relevant to acknowledge and explore the participants’ practices of sociality, such as drug giving and sharing.

The moral economy of sharing in drug cultures is well documented by the ethnographic work of Bourgois [19,20] and the act of giving heroin in 'a community of addicted bodies’ is based on a moral value of reciprocity [21]: *“It is considered unethical to leave a person stranded when he or she is dopesick , unless one is openly feuding with that person”*. We suggest that such communities are also communities of 'knowers’ – namely, people who possess corporeal knowledge of withdrawal and are thus, quite literally, able to understand the corporeal condition of another human being in that state. Importantly, the act of giving to a friend in withdrawal with known tolerance for opioids may have a lower harm potential than that of selling to unknown and potentially opioid-naive persons [4].

When a heroin-dependent individual is included in OMT, he or she might struggle to navigate norms of different social systems: the treatment system with its external control measures and possibilities for 'sanctions’ for what is perceived as 'diversion’ and drug using communities with their values of civil and informal execution of what is perceived as 'sharing and giving’. The logics upon which these systems are based – namely, 'harm’ and 'helping’, respectively – might work against each other. OMT enrollment can hence place someone in a position in which he or she violates his or her norms of helping and sharing in order to follow treatment regulations. Diversion’, as this term is conventionally interpreted by clinicians, is not typically understood as practices of giving and helping. These practices may nevertheless be perceived as such by those who undertake them. For those who participated in this study, it was not as though norms for interpersonal relations maintained while using illegal drugs could be nullified when entering treatment. As we see it, the need to sustain oneself as a decent person in one’s own eyes and those of others should be recognized. Thus, the 'positive’ interpersonal skills and practices of OMT patients could perhaps be further explored as possible resources throughout the clinical encounter and rehabilitation process. Further, clinicians should encourage and support strategies developed by individuals in OMT to avoid diversion of their opioid prescriptions, such as changing from one dispensing pharmacy to another in an effort to avoid potential buyers in certain areas. Finally, treatment providers should focus on the patients’ social lives and indeed consider including couples if both individuals are motivated for starting OMT.

## Abbreviations

OMT: Opioid maintenance treatment.

## Competing interests

The authors declare that they have no competing interests.

## Authors’ contributions

TC was project manager. TC, IH and ALM conceived the qualitative project and constructed the interview guide. IH conducted the interviews. IH and ALM conducted the analysis and wrote up the first draft of the paper. All authors contributed to and have approved the final manuscript.
